# Needs for Aging in Place: Views of Older Moroccan Adults in the Netherlands

**DOI:** 10.1093/geront/gnad154

**Published:** 2023-11-06

**Authors:** Hager Hussein, Jane Murray Cramm, Anna Petra Nieboer

**Affiliations:** Department of Socio-Medial Sciences, Erasmus School of Health Policy & Management, Erasmus University Rotterdam, Rotterdam, South Holland, The Netherlands; Department of Socio-Medial Sciences, Erasmus School of Health Policy & Management, Erasmus University Rotterdam, Rotterdam, South Holland, The Netherlands; Department of Socio-Medial Sciences, Erasmus School of Health Policy & Management, Erasmus University Rotterdam, Rotterdam, South Holland, The Netherlands

**Keywords:** Age-friendly community, Migrants, Neighborhood resources, Person–environment fit, Q methodology

## Abstract

**Background and Objectives:**

Due to health and/or financial limitations, older migrants may become especially dependent on their neighborhoods, highlighting the importance of investigating their experiences. We explored older Moroccan adults’ views on the relative importance of neighborhood resources for aging in place.

**Research Design and Methods:**

Thirty Moroccans aged ≥65 years residing in Amsterdam, Rotterdam, The Hague, and Utrecht were interviewed and asked to perform a ranking task developed with the combined quantitative and qualitative Q methodology. They ranked the relative importance of 38 statements representing the World Health Organization’s 8 global age-friendly cities domains, with explanation of their reasoning. By-person factor analysis was performed to identify factors representing distinct viewpoints, which were interpreted with reference to the interviewees’ comments.

**Results:**

Four viewpoints were identified: “home sweet home”; “connected, well-informed, and engaged”; “suitable and affordable living”; and “a lively neighborhood.” The perceived importance of neighborhood resources for aging in place differed among viewpoints.

**Discussion and Implications:**

Older Moroccan adults prioritize different neighborhood resources for aging in place. Our findings suggest that their diverse needs can be satisfied by enabling family to live in close proximity, providing diverse, inclusive neighborhoods with affordable, suitable housing, understandable information, social/cultural activities, and care services for vulnerable groups. Future studies may build on our findings to explore older (migrant) adults’ views on needs for aging in place in the Netherlands and other western countries.

## Background

Rapidly growing aging urban populations are increasingly ethnically diverse (van der Greft & Fortuijn, 2017). In 2019, 12% of the global migrant population was aged ≥65 years and 15% resided in developed areas (United Nations Department of Economic and Social Affairs, Population Division, 2019). Most western governments expected that older migrants would return to age in their home countries, but most, including Moroccans in the Netherlands (Schans, 2008), are staying in their host countries (Ciobanu et al., 2017).

Moroccans form one of the largest non-European migrant groups in the Netherlands (de Regt et al., 2022). In 2019, 13% of this group was aged ≥55 years (average, 66 years; 20% aged ≥75 years), 45.3% was female, and 18.6% lived alone (de Regt et al., 2022). Most migrated from rural areas of Morocco in the 1960s as “guest workers” because of a domestic labor scarcity, and have limited education (>80% of men and >90% of women did not complete elementary school; Ciobanu et al., 2017; Schellingerhout, 2004). More Moroccans came to the Netherlands in subsequent decades for family forming and reunification (Stock, 2014). These migrants now age in places that differ markedly—socioculturally and linguistically—from their home country (Pot et al., 2020); many older Moroccan women (70%) and men (31%) have difficulty conversing in Dutch (Schellingerhout, 2004).

Environmental gerontology has been used to identify neighborhood characteristics pertaining to age-friendliness for the facilitation of aging in place (Meeks, 2022; Spring, 2018; Van Dijk et al., 2015), defined as “the ability to live in one’s own home and community safely, independently, and comfortably, regardless of age, income, or ability level” (Centers for Disease Control and Prevention, 2022). The ecological theory of aging (Lawton & Nahemow, 1973) holds that (resources of) older adults, resources in their environments, and aging in place are interdependent, with vulnerable (financially, socially, and/or mobility-limited) groups tending to depend more than others on their environments (Iwarsson et al., 2007; Van Dijk et al., 2015). Thus, exploration of the degree of fit between environmental resources and older adults’ abilities and needs, rather than the examination of these aspects separately, is crucial (Iwarsson et al., 2007). The person–environment fit is key for successful adaptation across the lifespan (Baltes, 1987; Wahl & Gerstorf, 2020; Wahl & Weisman, 2003). Diversity is especially important for older adults, to account for cultural and economic inequities in resource access and influences on the ability to age in place (Meeks, 2022).

Neighborhood (physical, social, and municipal) resources determine older adults’ ability to realize well-being and age in place. Personal resources influence aging-in-place abilities and may account for differences in needs (Nieboer & Cramm, 2022). Older Moroccan women, for example, tend to spend more time at home and have fewer social networks than do men; they are expected to perform (sometimes burdensome) tasks such as caring for grandchildren, and have difficulty refusing when they feel unable (Omlo et al., 2016). The living situation (alone/with others) influences older adults’ independence and choice of informal (spouse-/child-provided) or professional care (van Hoof et al., 2022). Older Moroccans depend strongly on their children as main sources of informal care and support, including when they can no longer take care of themselves or a partner dies (Omlo et al., 2016).

(Non-European) migrants in the Netherlands tend to concentrate in certain neighborhoods; 38% of Moroccan migrants reside in ≥50% migrant-populated neighborhoods (Gijsberts & Dagevos, 2005). Many such neighborhoods are deprived, limiting personal resources and societal integration and increasing social inequality and exclusion (Van der Greft et al., 2016). Although 56% and 61% of older Moroccan men and women feel at home in the Netherlands, 51% and 41%, respectively, perceive discrimination against them (Schellingerhout, 2004). Such conditions impose physical and social constraints that impair well-being realization (Lager et al., 2012) and successful aging in place (Cramm et al., 2018). Due to health and/or financial limitations, older migrants may become especially neighborhood-dependent (Buffel, 2017).

Like older Dutch adults, older migrants prefer to live independently in their homes as long as possible (Witter & Fokkema, 2018). The Dutch government, municipalities, and social organizations have made numerous efforts to promote aging in place, collaborating to improve older adults’ living/neighborhood conditions, home care, and independent living ability (Ministry of Health, Welfare and Sport, 2018; Van Triest & Van Vliet, 2017). Small-scale initiatives directed toward older migrants have culture-specific housing and care components, including daycare facilities that serve as meeting places and sources of support and health-related information (Witter & Fokkema, 2018).

This study was conducted to explore the views of older Moroccan adults in the Netherlands on the relative importance of neighborhood resources for aging in place using Q methodology, which enables the study of individuals’ beliefs, experiences, and perspectives (Watts & Stenner, 2012). We aimed to contribute to the promotion of more-inclusive aging-in-place policies that meet the demands of diverse groups of older adults, in line with critical foci of efforts in this field (van Hoof et al., 2022).

## Method

### Participants

The study was part of broader research on community age-friendliness and well-being realization among older natives and Moroccan, Turkish, and Surinamese migrants in the Netherlands (Nieboer & Cramm, 2022). It was conducted with first-generation Moroccans aged ≥65 years living, as do 44.9% of Moroccan migrants (Statistics Netherlands, 2022), in the four largest cities in the Netherlands (Amsterdam, Rotterdam, The Hague, and Utrecht; Supplementary Material Section 1). These migrants preferred to settle in these cities on arrival because of better job opportunities and the presence of other Moroccans, and they prefer to continue living there (van der Star et al., 2021). We strove to include participants from diverse neighborhoods residing in the Netherlands for ≥6 months/year.

With the help of social workers and volunteers who worked with this population, participants were recruited via social networks, neighborhood center and mosque visitation, and flyer distribution in mailboxes and Moroccan shops. Participants received 30-euro gift vouchers. The research ethics committee of Erasmus University Rotterdam approved the study (ETH2122-0125).

### Q Set

In the larger study, a representative 38-statement Q set was developed to identify participants’ viewpoints on the relative importance of neighborhood resources for aging in place (Nieboer & Cramm, 2022; Supplementary Material Section 2). The statements fell into eight World Health Organization (2007) global age-friendly cities domains: outdoor spaces and buildings, transportation, housing, social participation, respect and social inclusion, civic participation and employment, communication and information, and community support and health services.

### Interviews and Q-Sort Procedure

**Figure 1.**
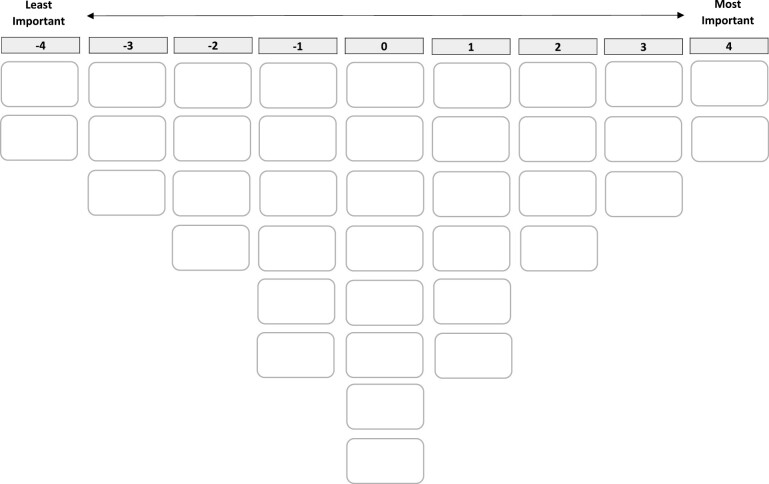
Sorting grid.

Interviews (average, 50 min) were conducted at neighborhood centers or participants’ homes, in Arabic and/or Dutch by the first author and in Berber by another interviewer, according to participants’ preferences and/or proficiency. A Dutch interview guide was used to ensure standardization (Supplementary Material Section 3). The interviewer introduced the study and tasks and obtained the interviewee’s consent to participate with audio-taping. Then, she presented printed cards with the Q-set statements in easy-to-read Dutch or Arabic text to the interviewee, asking him/her to read them and place them in three piles according to importance (important, less/not important, neutral) for feeling good in the neighborhood. The participant was then asked to rank the statements in each pile from most (+4) to least (−4) important on a sorting grid (Figure 1), and to elaborate on the rankings (Supplementary Material Section 4).

After the interview, the participant filled out a questionnaire, providing information about their age, gender, postal code, living situation (alone/with others), health condition (number of chronic diseases in the last 12 months, walking and vision problems), highest educational level (seven categories ranging from <6 years of elementary school to university or higher education, total number of years in school), and Dutch proficiency (do not speak or often/sometimes/never have difficulty speaking; the first category was taken to reflect difficulty speaking Dutch). The interviewers transcribed the audio recordings and translated the transcripts into English.

### Analysis

The Q sorts were entered into PQ Method 2.35 (Schmolck & Atkinson, 2002). A correlation matrix was calculated and subjected to centroid factor analysis to extract factors of interest. The factors were subjected to varimax rotation, and an idealized Q sort was computed for each. Qualitative interview data were used to clarify the reasons underlying participants’ statement rankings. Viewpoint interpretation is detailed in Supplementary Material Section 5.

## Results

Of 36 individuals who consented to participate in the study, six were excluded due to difficulty understanding the tasks (*n* = 3), dementia (*n* = 1), and incomplete Q-set ranking (one statement missing; *n* = 2). Thus, the sample comprised 30 participants (mean age, 70 years; 60% women, 50% living alone, 50% with no education; Table 1).

**Table 1.** Background Characteristics of the Study Participants (*n* = 30)

No.	Age (years)	Gender	Education	Years of education	City	Living situation	Health conditions			Language of interview
							Multimorbidity^a^	Walking problems	Vision problems	
1	72	M	High school	11	Rotterdam	With others	No	No	No	Arabic
2	66	F	<Elementary school	4	Rotterdam	Alone	Yes	Yes	Yes	Arabic
3	77	M	Elementary school	NR	Amsterdam	With others	No	Yes	No	Dutch
4	85	M	Elementary school	10	Amsterdam	With others	No	Yes	No	Dutch
5	74	M	Elementary school	4	Amsterdam	With others	Yes	No	No	Arabic
6	70	F	No education	0	Amsterdam	With others	Yes	Yes	No	Arabic
7	67	F	Elementary school	7	Amsterdam	With others	No	No	No	Arabic
8	69	M	University	24	Rotterdam	Alone	No	No	No	Dutch
9	65	F	<Elementary school	7	Utrecht	Alone	Yes	Yes	No	Arabic
10	76	M	<Elementary school	NR	Utrecht	With others	Yes	Yes	Yes	Arabic
11	83	F	No education	0	Rotterdam	With others	Yes	Yes	No	Berber
12	65	F	No education	0	Rotterdam	Alone	No	No	No	Berber
13	67	F	No education	0	Rotterdam	Alone	Yes	No	No	Berber
14	71	F	No education	0	Utrecht	Alone	Yes	Yes	Yes	Arabic
15	70	F	No education	0	Utrecht	Alone	Yes	Yes	No	Berber
16	75	F	No education	0	Utrecht	Alone	Yes	Yes	No	Berber
17	71	F	No education	0	Utrecht	With others	Yes	Yes	Yes	Berber
18	70	F	<Elementary school	1	The Hague	Alone	No	Yes	No	Berber
19	73	M	Higher education	16	Rotterdam	With others	No	No	No	Dutch
20	67	M	No education	0	Rotterdam	With others	Yes	Yes	No	Berber
21	68	M	No education	0	Rotterdam	Alone	Yes	Yes	Yes	Arabic
22	64^b^	M	<Elementary school	3	Rotterdam	With others	Yes	Yes	Yes	Arabic
23	72	F	No education	0	Rotterdam	With others	Yes	Yes	Yes	Arabic
24	66	F	<Elementary school	NR	Amsterdam	Alone	Yes	No	No	Berber
25	77	M	No education	0	Amsterdam	With others	No	No	No	Dutch
26	68	M	Higher Education	15	Rotterdam	Alone	Yes	Yes	No	Dutch
27	66	F	No education	0	Rotterdam	Alone	Yes	Yes	No	Berber
28	72	F	No education	0	The Hague	With others	No	Yes	Yes	Dutch
29	68	F	<Elementary school	5	The Hague	Alone	Yes	Yes	No	Arabic
30	67	F	No education	0	The Hague	Alone	Yes	Yes	No	Arabic

*Notes*: F = female; M = male; NR = not reported.

^a^Having two or more chronic diseases.

^b^This participant was interviewed 2 weeks before his 65th birthday because of his travel plans.

The analysis revealed four distinct viewpoints (factors) that explained 42% of the data variance (Table 2). The Q sorts of 26 participants associated significantly with one of these factors.

**Table 2.** Idealized Q Sorts for the Factors (Viewpoints)

WHO domains and statements		Viewpoints		
	Factor 1	Factor 2	Factor 3	Factor 4
Outdoor spaces and buildings				
1. A clean and well-maintained neighborhood.	1	2	1	1
2. Plenty of green.	3	2	−3**	2
3. Benches.	2**	−1	−2	0
4. Good sidewalks and crosswalks.	2**	0	0	0
5. A safe neighborhood.	3	0*	2	2
6. Accessible buildings.	1	−2*	−1	0
7. No nuisance.	2	1	1	1
8. Public toilets.	−4	0	−4	2
9. Beautiful buildings.	−2	−3	−2	0**
Transportation				
10. Good public transport.	−1	0	0	4**
11. Special transport for older adults with disabilities.	0	0	0	−1
12. Sufficient parking spaces.	0	−3*	1	−1*
13. Cycling and walking trails.	1	−2	−3	2
Housing				
14. Affordable housing.	2	4	3	0**
15. Suitable homes for older adults.	0	−1	4**	0
Social participation				
16. A neighborhood where social/cultural activities are organized.	−3**	1	−1	0
17. Affordable activities.	−2	0**	−2	−4
18. A meeting place for older adults.	−2	−1	3**	−1
19. Activities especially for Moroccan people.	1	0*	2	−2*
Respect and social inclusion				
20. A neighborhood where people have respect for older adults.	1	0	0	3
21. A neighborhood where people know each other.	0*	4**	2	1
22. Friends and/or family in the neighborhood.	4	−4**	0**	4
23. A neighborhood with people from the same background.	0**	−4	−4	−4
24. No discrimination in the neighborhood.	1	3*	1	−1**
25. Contact between young and old in the neighborhood.	−1**	2	1	1
Civic participation and employment				
26. Opportunities to volunteer.	−3*	1**	−3	−1
27. A neighborhood where older adults have a say.	−2	−1	3	0
28. Availability of courses or trainings in the neighborhood.	−3**	1**	−1	−1
Communication and information				
29. Understandable information about services and activities in the neighborhood.	0*	3**	−1	−2
30. Municipal information in a central place	−1	−2	−1	−3
31. A neighborhood where people keep each other informed about what happens.	−1	3**	−1	−2
Community support and health services				
32. A neighborhood where home care is easy to get.	0	−2	0	−3
33. A neighborhood where care providers work together and inform each other.	−1**	1	0	1
34. Family doctor and pharmacy in the neighborhood.	4	−1**	4	3
35. A place where I can go for advice and support.	0	−3*	0	−2
36. Volunteers who provide assistance when needed.	−1	2**	−2	−3
37. Shops and other amenities in the neighborhood.	3*	1	2	1
38. Opportunities for sports in the neighborhood.	−4**	−1**	1**	3**

Notes: WHO = World Health Organization.

*p < .05, **p < .01, distinguishing statements for the factors.

### Viewpoint 1: Home Sweet Home

Nine participants had this viewpoint; eight were women, seven lived alone, six had no education, six had difficulty speaking Dutch, seven had multimorbidity, and six had walking problems. They visualized their neighborhoods as places supporting a quiet home life, highlighting the importance of family members’ presence and the availability of shops and health services within walking distance.

Participants preferred to stay in their cozy home environments. Especially given their difficulty speaking Dutch, they depended on children and grandchildren [Q-set statement (S) 22: +4]. For them, family members were companions who helped to combat loneliness and provided care, safety, and support:

I moved last year and now I live one street behind my daughter. In that way, I get the necessary help and companionship … I honestly wouldn’t even want to live without family around me, existence would be too lonely then. Also, because I can’t communicate in this country, it’s more difficult. My children and grandchildren arrange all my appointments, errands, and everything for me. [Participant 11]

These participants gave less importance to interaction with others in the neighborhood (S21: 0; S25: −1), and preferred that any such interaction was with Moroccans (S23: 0). They did not find neighborhood opportunities for social/civic/sports participation to be important (S16: −3; S17: −2; S18: −2; S26: −3; S27: −2; S28: −3; S38: −4):

We aren’t used to going to all kinds of social activities. We prefer to sit quietly at home because we’ve always lived that way. Our husbands used to be strict, you couldn’t just go anywhere. Even though my husband passed away, I still live like that. I just don’t have the need. [Participant 15]

They wanted to live in green neighborhoods that they could enjoy from their windows (S2: +3):

I think it’s important that there is enough greenery in my surroundings. I really enjoy looking out my window at greenery … Especially since I can’t go outside much, I want it close to me … [Participant 11]

Given their health issues, participants emphasized the importance of having general practitioners (GPs) near their homes (S34: +4). Despite their dependence, they did not want to constantly burden family members and wanted to live in neighborhoods that supported their independence (e.g., ability to occasionally shop alone). Thus, they appreciated the availability of shops, especially ethnic supermarkets/bakeries and halal butchers, within walking distance (S37: +3):

That way I can go alone every now and then. If it’s far, my daughter will always have to take me. That would be unpleasant. [Participant 13]

To remain independent (including after being widowed), these participants needed safe crosswalks, accessible buildings, and benches (S4: +2; S6: +1; S3: +2) in safe neighborhoods that allowed them to go out without fear (S5: +3):

Because I live alone, I think a safe neighborhood is very important. I was used to living with ten children and a husband and it’s very different now. Now I’m much more aware of my safety, and I get frightened more often. [Participant 12]

As these participants stayed home and prioritized service proximity, public transport and toilets in the neighborhood were not important to them (S10: −1; S8: −4). The latter also reflects religious practice:

I’d rather hold it till I go home than do it while out … We’re Muslims and of course we wash ourselves as soon as we go to the toilet. It’s just not pleasant to go to the toilet while out. [Participant 15]

### Viewpoint 2: Connected, Well-Informed, and Engaged

Four men who lived with their partners and/or children and had completed elementary school or less held this viewpoint; three participants each spoke Dutch, had multimorbidity, and had walking problems. They prioritized tolerance, social connection/engagement, and affordable housing in their neighborhoods.

Participants felt that tolerance and respect for everyone in the neighborhood (S24: +3) was required for the forging and maintenance of social connections:

There are different people, different colors … but respect is always good … Respect as everyone has their rights. Don’t discriminate against anyone. [Participant 3]

These participants were against segregated neighborhoods (S23: −4), emphasizing that neighborhood diversity enriches information exchange and creates opportunities to learn about other cultures, norms, and traditions:

When you see different people you can experience more things, but when you’re locked in then nothing happens … The more people there are the more you get of everything … [Participant 3]

Participants desired to live in cohesive neighborhoods with supportive neighborly relationships (S21: +4):

We have a saying …: your near neighbor is better than your distant brother. So, you must have good contact with people. [Participant 3]

They felt that contact between younger and older generations was important (S25: +2):

When the person is generally in contact with younger and older adults, they benefit from each other; the older benefits from the young and the young benefits from older ones. [Participant 10]

These participants wanted to stay engaged and valued opportunities for social/civic participation (S16: +1; S17: 0; S26: +1; S28: +1). They valued information about activities and services and felt that it should be understandable for everyone (S36: +2) and readily available in the neighborhood (S29: +3). They kept updated on relevant information mainly through neighbors (S31: +3); seeking information about advice and support (S35: −3) and the availability of municipal information in a central place (S30: −2) were less important.

These participants highly valued the neighborhood availability of affordable housing (S14: +4):

This is a must. People are vulnerable … Now I fall under the National Old Age Pensions Act. When you finish your 65 years, they give you 800 or 900 euro … The housing prices are getting to be troublesome. [Participant 5]

As these participants lived with others and relied on good neighborly connections, they did not require extra help at home (S32: −2) or other family members in the neighborhood (S22: −4). They also found neighborhood characteristics such as safety, beautiful and accessible buildings, and transport to be less important (S5: 0; S9: −3; S6: −2; S12: −3; S13: −2).

### Viewpoint 3: Suitable and Affordable Living

Nine participants had this viewpoint; five were men, six lived with partners and/or children, five had no education, six spoke Dutch, four had multimorbidity, and six had walking problems. They prioritized good-quality housing, indoor meeting places, and health services within walking distance in their neighborhoods.

Participants emphasized the need for affordable age-friendly homes with accessible amenities (S15: +4; S14: +3):

For the houses to be “age-proof,” meaning that there’s an elevator, that the house doesn’t have many stairs or is on the ground floor if possible … It’s important in the sense that older adults also have an opportunity to live in the same neighborhood where … they’re used to living. [Participant 19]

Participants wanted to be heard and have their complaints taken seriously (S27: +3). They criticized housing organizations due to the difficulty of finding more-suitable homes when needed. They described often having very limited housing options located outside the city:

I’ve been trying to get another house for a long time and I don’t get a chance (in the social housing program). I’m not the only one, I hear it from other people as well … I think this is the most important thing, the municipality must help people with this. [Participant 25]

They expressed that new, “better” homes are too expensive for older adults, given their limited financial resources:

Almost all people are retired, and their income is low … Old houses have an affordable rent, but the newly built houses are … very expensive! [Participant 1]

These participants prioritized health service proximity (S34: +4) due to their health issues. As they aged, they depended more on the neighborhood availability of GPs:

The older you get, the more complaints you get … the possibility to see the doctor quickly if you need, or that the doctor comes quickly to you if something is wrong, must be there. So, depending on the seriousness of the complaints you have, it’s important that the doctor is nearby. [Participant 19]

Limited by health issues, participants were not interested in activities (S16: −1; S17: −2; S26: −3; S28: −1) or cycling (S13: −3). They preferred indoor gatherings with similarly aged people (S18: +3), and gave less importance to outdoor green areas, benches, and public toilets in the neighborhood (S2: −3; S3: −2; S8: −4). They valued indoor meeting spaces where they could see familiar faces and converse (S19: +2):

An older person doesn’t work anymore. He stays at home and when he goes out, he looks for where he feels at ease with friends who speak his language … [Participant 1]

Although these participants preferred to interact with speakers of their language, they perceived neighborhood segregation as discriminatory and a source of conflict (S23: −4). They noted that Islam acknowledges that God created people of different races and tribes and promotes knowing and respecting one other:

They’ve done that *[neighborhood segregation]* a few times, but it created conflict ... It’s better for the neighborhood to be mixed, then there’ll be no conflict … To me this is discriminating … why do we have to live separately? [Participant 25]

### Viewpoint 4: A Lively Neighborhood

Four participants had this viewpoint: two were men who lived alone and had university/higher education, three spoke Dutch, and two had multimorbidity and difficulty walking. They viewed neighborhoods as places that encourage older adults to remain socially and physically active, with health services available.

Participants prioritized having friends in the neighborhood (S22: +4) who motivated them to maintain social lives, and did not need extra care services (S32: −3; S36: −3):

This is the most important thing for me, to have friends … neighbors … to have company. We can go out together, gather … relieve stress with each other … Here, I have neighbors, I have old friends, and we get along with each other [Participant 7]

These participants wanted to engage in social and cultural activities more than did participants with Viewpoints 1 and 3 (S16: 0), and activity affordability was not an issue (S17: −4). They aspired to have diverse, not exclusively Moroccan, social networks (S19: −2). They recognized that discrimination could not be avoided, but preferred to not focus on it (S24: −1). They were against segregation (S23: −4):

If I were a municipal housing officer, I’d never put people of one descent in one corner because then you will make a ghetto, and we’ve seen what ghettos have created in America … [Participant 26]

As these participants had health issues, they appreciated having GPs in the neighborhood (S34: +3). They wanted their neighborhoods to stimulate and facilitate their mobility, with opportunities for sports (S38: +3) and good transportation options (S10: +4; S13: +2):

I have a free transportation card … it’s very important for me because then I can go places where I remain a bit active or … where I also meet people … [Participant 26]

As these participants were active and spent time outside, public toilets and beautiful buildings in the neighborhood were more important for them than for participants with other viewpoints (S8: +2; S9: 0). Given their proactive attitudes and high educational levels, they did not depend on their neighborhoods to acquire information (S29: −2; S30: −3; S31: −2). They obtained information about activities and services and arranged their affairs online:

Nowadays, we have Google, we can find everything … information doesn’t necessarily have to be in a central city hall … [Participant 8]

### Consensus Among Viewpoints

**Figure 2.**
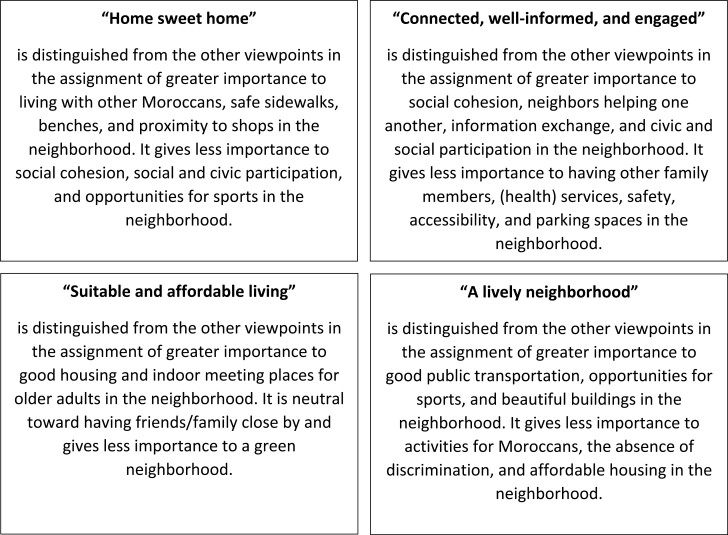
Distinguishing the identified viewpoints.

The viewpoints identified are distinct (Figure 2), with some similarities. Participants with Viewpoints 1–4 recognized the importance of clean, well-maintained (S1: +1, +2, +1, +1; see Author Notes), quiet (S7: +2, +1, +1, +1) neighborhoods, as they had become less tolerant of nuisance with age. To some extent, they valued respect for older adults (S20: +1, 0, 0, +3), and indeed everyone, in their neighborhoods.

## Discussion and Implications

Research has revealed variability in (frail) older adults’ views on ideal neighborhood characteristics for aging in place (Van Dijk et al., 2015). Similarly, Conkova and Lindenberg (2020) found differences within and among older migrant groups’ experiences of aging and needs for aging well, related to gender, educational background, migration history, job history, and socioeconomic status. Building on these findings and given the need for research to inform the provision of more-inclusive aging-in-place policies (van Hoof et al., 2022), we explored the views of older Moroccans in the Netherlands and identified four viewpoints reflecting their diverse needs.

Most participants with Viewpoint 1 were women who spoke little Dutch, lived alone, and preferred quiet home lives and interaction with only family members and Moroccans. These results support previous findings that older migrants generally depend on family members in the same neighborhoods as their main social networks, and the presence of people with the same backgrounds to have a sense of home (Buffel & Phillipson, 2011). Most older Moroccan women came to the Netherlands at older ages than their husbands, and largely stayed at home to care for children, which limited their social networks and learning of Dutch. Thus, they have more difficulty arranging care, housing, and social participation (Verkaik et al., 2019). The longer life expectancy of (Moroccan) women increases the risk of living without a partner longer, which together with the language barrier increases the chance of high vulnerability (Omlo et al., 2016). Given that older migrants without nearby family or friends, especially those with functional and/or resource limitations, are more prone to loneliness, social isolation, and depression (Buffel & Phillipson, 2011), older Moroccans (especially women) could benefit greatly from policies that enable (extended) family members to live in close proximity, supporting their roles as companions and informal caregivers.

Participants with Viewpoint 2 were men who prioritized cohesive, inclusive neighborhoods. Many first-generation migrants in the Netherlands are less satisfied than Dutch natives with the quality of their social relationships (despite similar contact frequency), leading to greater loneliness and possibly explaining this preference (Ten Kate et al., 2020). Older migrant men in the Netherlands tend to have more opportunities than do women to combat loneliness and expand their networks by attending social/cultural/religious activities in neighborhood centers and mosques (perceived male spaces; Omlo et al., 2016). Most participants holding this viewpoint spoke Dutch, which may facilitate the building of neighborly relationships, especially with Dutch natives, and participation in activities (Omlo et al., 2016). However, many migrants in the Netherlands feel unaccepted and unwelcomed by Dutch natives (Andriessen et al., 2020), emphasizing the need for policies promoting older migrants’ social inclusion. Participants with this viewpoint also valued the neighborhood availability of understandable information; in contrast, those with Viewpoint 4 were more educated and preferred to obtain information online. Thus, the means by which (neighborhood-relevant) information is provided in the Netherlands should account for older Moroccans’ educational levels.

Participants with Viewpoint 3 prioritized the neighborhood availability of good-quality, affordable housing. Provisions to make homes suitable and accessible for older adults (e.g., handrail/bathroom grab bar installation) reduce the risk of injury and promote independence and well-being maintenance (Mulliner et al., 2020). Research has shown that older (Moroccan) migrants, like our participants with Viewpoints 2 and 3, are more likely than Dutch natives to live with partners and/or children (who may not have separate incomes), which may impose an extra financial burden and reduce their standard of living (van der Greft & Fortuijn, 2017). This may account for these participants’ major concern about housing affordability, suggesting that its lack could threaten their ability to age in place. In line with previous findings, participants with Viewpoint 3 appreciated the availability of public meeting places, on which older (especially male) migrants depend to maintain contact with people with the same background and feel at home (Buffel, 2017).

Participants with Viewpoint 4 emphasized the importance of friends, good public transport, and sports opportunities in the neighborhood to facilitate their social and physical activities, suggesting that neighborhood physical and social characteristics are closely related and supporting Levasseur et al.’s (2015) finding that public transport availability is associated with older adults’ mobility and social engagement. In addition, Van Cauwenberg et al. (2014) showed that increased neighborhood social connections encouraged older adults to improve their physical activity, as they more often chose walking as transportation. Research suggests that older migrants make efforts to build relationships with neighbors, aspiring to include people from different backgrounds on whom they can rely for help and social support, thereby maintaining resilience (Klokgieters et al., 2020). Half of the participants with this viewpoint had received higher education, associated with greater satisfaction with physical activity and social participation and easier maintenance of contact with friends and relatives (Verkaik et al., 2019).

Participants with three of the four identified viewpoints wished to live in diverse neighborhoods and perceived segregation as discrimination. Participants with Viewpoint 3 might prefer to connect with other Moroccans (like those with Viewpoint 1), but these three viewpoints generally reflect older Moroccans’ aspiration to establish diverse networks, which is discrepant from their reality. Previous research has yielded mixed findings; Boileau et al. (2022) described segregation as a “mixed bag” with potentially negative and positive outcomes. Bécares et al. (2018) observed that segregation can protect against discrimination and enhance social support from people with similar backgrounds (consistent with Viewpoint 1), whereas Gijsberts and Dagevos (2005) demonstrated that segregated neighborhoods force connection only with people with the same ethnic background, reducing mutual acceptance. Thus, most older Moroccans’ wish to live in diverse neighborhoods is understandable; it could help them diversify their social networks, increase mutual acceptance, and improve their Dutch proficiency (Gijsberts & Dagevos, 2005).

Our findings have implications for policymakers, as they highlight neighborhood characteristics needed to support older (Moroccan) migrants’ aging in place in the Netherlands. For those with Viewpoint 1, who may impose pressure on their children (Choi et al., 2023), policies are needed to support the children’s role as informal caregivers, with options for relocation to the parents’ neighborhoods when needed. The Dutch government encourages citizens to provide informal care, and views family members as the first line of care provision to older adults; it provides support (flexible working hours, subsidies, and substitute caregivers when needed) to reduce informal carers’ burdens (Vos et al., 2021). Aging-in-place policies need to support training to enhance the cultural competence, sensitivity, and understanding of diverse care needs of those who provide home-based care to older migrant adults. For older Moroccans with Viewpoint 2, policies and programs are needed to promote social inclusion through the development and maintenance of diverse social-support networks and provision of opportunities for social interaction, cultural preservation, and experience sharing. Consideration must be given to the inclusion of vulnerable older adults, like those with limited (financial) resources, widows/widowers, and non–Dutch speakers. Older Moroccans with Viewpoints 2–4 would benefit from efforts to create and maintain diverse and inclusive neighborhoods, promoting connection among (older) adults with different backgrounds. This is not easy, as the issue of ethnic segregation has received public and political interest in the Netherlands, as in other western countries. The development of policies aiming to reduce ethnic segregation is inconsistent with the country’s constitution, which forbids discrimination (i.e., distinction between citizens based on race, country of origin, or minority identity; Boterman et al., 2021). Many policies aiming to socioeconomically improve deprived neighborhoods through house renovation and the attraction of middle-/high-income individuals have been implemented (Gijsberts & Dagevos, 2005), but housing affordability (especially important for older Moroccans with Viewpoints 2 and 3) should not be compromised. Policies could encourage larger-scale collaboration among sectors and organizations involved in aging, healthcare, immigration, and social services, ensuring a holistic, integrated approach to meet the diverse needs of older (migrant) adults while avoiding fragmentation and improving service delivery (Black & Oh, 2023).

Our findings indicate that (Moroccan) migrant groups should not be perceived as homogeneous, and that a “one-size-fits-all” approach to policy/intervention implementation to improve aging in place is not suitable, in the Netherlands or throughout the western world, where migrant populations are increasing annually. Migration trends have changed western countries’ population compositions, with far-reaching and local implications for the aging experience (Meeks, 2020). More research is needed to explore the diverse needs of older (migrant) adults for aging in place, with the consideration of differences within and among groups to inform the implementation of more-inclusive policies and interventions that satisfy these needs.

### Strengths and Limitations

A study strength is the use of Q methodology, which enables the exploration of different viewpoints, understanding, and comparing them, without requiring large participant samples (Brown, 1980; Watts & Stenner, 2012), and is thus promising for research conducted with difficult-to-recruit older migrants (Bilecen & Fokkema, 2022). Additionally, Q methodology provides a flexible approach for the study of subjectivity to derive meaning from participants’ individual experiences (Brown, 1980). For example, Q-set items can be adjusted to suit target populations, for example, through the use of clear (translated) text or imagery (Brown, 1980; Supplementary Material Section 2); this approach is thus suitable for vulnerable populations (Combes et al., 2004). Such flexibility supported our efforts to represent the views of individuals with little to no education; most of our participants did not read well. The interviewers repeatedly read the statements out loud in the interviewees’ preferred language and gave participants more time when needed to rank the statements. We believe that participants were able to express their views strongly, as reflected in discussions about important topics such as family members’ roles as informal caregivers, the housing crisis, discrimination, and ethnic segregation in the Netherlands. Study limitations are that our sample is not representative of all older Moroccans in the Netherlands, and the results obtained with such Q methodology application are not generalizable (Watts & Stenner, 2012). Surveys are needed to explore the prevalence of the identified viewpoints in the country’s general older Moroccan population. Additionally, the four viewpoints explained 42% of the variance, common in Q methodological research (Watts & Stenner, 2012) and reflecting diversity in aging-in-place needs, as in previous research (e.g., Van Dijk et al., 2015). Future studies could build on our findings, exploring relevant views among diverse older-adult groups in the Netherlands. Given that neighborhood characteristics differ among cities and countries, the perspectives of older (migrant) adults living in diverse contexts also need to be explored. Quantitative research on whether (and to what extent) participants’ available neighborhood resources influence their views on aging in place is needed. Finally, we focused only on Moroccans aging in the Netherlands; comparison with the views of Moroccans aging in Morocco (among the worst countries for aging in place due to socioeconomic and elder/healthcare deficiencies; Abyad & Formosa, 2021) would be useful.

## Conclusion

Using Q methodology, we identified four viewpoints held by older Moroccans in the Netherlands on the neighborhood resources needed to age in place. Our findings increase the understanding of the person–environment fit, and the resources needed to facilitate older migrants’ aging in place. They illustrate the diversity of needs and unequal importance of neighborhood resources. This knowledge is crucial for policymakers who want to promote aging in place and make cities more age-friendly while considering the diverse needs of older (migrant) adults.

## Supplementary Material

gnad154_suppl_Supplementary_Material

## Data Availability

The data used in this study are currently (2023) unavailable to other researchers, as this research is part of an ongoing project entitled “Community age-friendliness and well-being realization among older natives and people with Moroccan, Turkish, and Surinamese backgrounds in the Netherlands” (Nieboer & Cramm, 2022). Data will be shared 1 year after the completion of the project (2027) upon reasonable request. This study was not preregistered.

## Author Notes

(S1: +1, +2, +1, +1) means that participants with Viewpoints 1, 3, and 4 ranked S1 (“A clean and well-maintained neighborhood”) as +1 and those with Viewpoint 2 ranked it as +2 (Table 2). Subsequent statements are structured in the same manner.

## References

[CIT0001] Abyad, A., & Formosa, M. (2021). Population ageing in the Middle-East and North Africa: Research and policy implications. United Nations International Institute on Ageing.

[CIT0002] Andriessen, I., Dijkhof, J. H., van der Torre, A., van den Berg, E., Pulles, I., Iedema, J., & de Voogd-Hamelink, M. (2020). Ervaren discriminatie in Nederland II [Perceived discrimination in the Netherlands II]. Sociaal Cultureel Planbureau. https://www.scp.nl/publicaties/publicaties/2020/04/02/ervaren-discriminatie-in-nederland-ii

[CIT0003] Baltes, P. B. (1987). Theoretical propositions of life-span developmental psychology: On the dynamics between growth and decline. Developmental Psychology, 23(5), 611–626. 10.1037/0012-1649.23.5.611

[CIT0004] Bécares, L., Dewey, M. E., & Das-Munshi, J. (2018). Ethnic density effects for adult mental health: Systematic review and meta-analysis of international studies. Psychological Medicine, 48(12), 2054–2072. 10.1017/S0033291717003580 29239292 PMC6076993

[CIT0005] Bilecen, B., & Fokkema, T. (2022). Conducting empirical research with older migrants: Methodological and ethical issues. Gerontologist, 62(6), 809–815. 10.1093/geront/gnac036 35303092 PMC9290876

[CIT0006] Black, K., & Oh, P. (2023). Exploring sectoral reach in age-friendly communities. Gerontologist, 63(5), 920–932. 10.1093/geront/gnac149 36183251

[CIT0007] Boileau, L. L., Bless, H., & Gebauer, J. E. (2022). The ‘mixed bag’ of segregation: On positive and negative associations with migrants’ acculturation. European Journal of Social Psychology, 52(3), 457–471. 10.1002/ejsp.2830

[CIT0008] Boterman, W. R., Musterd, S., & Manting, D. (2021). Multiple dimensions of residential segregation. The case of the metropolitan area of Amsterdam. Urban Geography, 42(4), 481–506. 10.1080/02723638.2020.1724439

[CIT0009] Brown, S. R. (1980). Political subjectivity: Applications of Q methodology in political science. Yale University Press.

[CIT0010] Buffel, T. (2017). Ageing migrants and the creation of home: Mobility and the maintenance of transnational ties. Population, Space and Place, 23(5), e1994. 10.1002/psp.1994

[CIT0011] Buffel, T., & Phillipson, C. (2011). Experiences of place among older migrants living in inner-city neighbourhoods in Belgium and England. Diversité Urbaine, 11(1), 13–37. 10.7202/1007742ar

[CIT0012] Centers for Disease Control and Prevention. (2022). Healthy places terminology. https://www.cdc.gov/healthyplaces/terminology.htm

[CIT0013] Choi, H., Reblin, M., & Litzelman, K. (2023). Conceptualizing family caregivers’ use of community support services: A scoping review. Gerontologist, 1–11. 10.1093/geront/gnad039 37022354 PMC11491529

[CIT0014] Ciobanu, R. O., Fokkema, T., & Nedelcu, M. (2017). Ageing as a migrant: Vulnerabilities, agency and policy implications. Journal of Ethnic and Migration Studies, 43(2), 164–181. 10.1080/1369183x.2016.1238903

[CIT0015] Combes, H., Hardy, G., & Buchan, L. (2004). Using Q‐methodology to involve people with intellectual disability in evaluating person‐centred planning. Journal of Applied Research in Intellectual Disabilities, 17(3), 149–159. 10.1111/j.1468-3148.2004.00191.x

[CIT0016] Conkova, N., & Lindenberg, J. (2020). The experience of aging and perceptions of “aging well” among older migrants in the Netherlands. Gerontologist, 60(2), 270–278. 10.1093/geront/gnz125 31565727 PMC7039376

[CIT0017] Cramm, J. M., van Dijk, H. M., & Nieboer, A. P. (2018). The creation of age-friendly environments is especially important to frail older people. Ageing & Society, 38(4), 700–720. 10.1017/s0144686x16001240

[CIT0018] de Regt, S., Fokkema, T., & Das, M. (2022). Migrantenouderen in Nederland: Een beschrijvende analyse van de leefsituatie van ouderen uit de 20 grootste herkomstgroepen [Older migrant adults in the Netherlands: A descriptive analysis of the living situation of older adults from the 20 largest ethnic groups]. Statistische Trends. https://www.cbs.nl/nl-nl/longread/statistische-trends/2022/migrantenouderen-in-nederland

[CIT0019] Gijsberts, M., & Dagevos, J. (2005). Uit elkaars buurt. De invloed van etnische concentratie op integratie en beeldvorming [Love thy neighbor? The influence of ethnic concentration on integration and interethnic attitudes]. Sociaal en Cultureel Planbureau. https://repository.scp.nl/handle/publications/1020

[CIT0020] Iwarsson, S., Wahl, H. W., Nygren, C., Oswald, F., Sixsmith, A., Sixsmith, J., Széman, Z., & Tomsone, S. (2007). Importance of the home environment for healthy aging: Conceptual and methodological background of the European ENABLE-AGE Project. Gerontologist, 47(1), 78–84. 10.1093/geront/47.1.78 17327543

[CIT0021] Klokgieters, S. S., van Tilburg, T. G., Deeg, D. J. H., & Huisman, M. (2020). The linkage between aging, migration, and resilience: Resilience in the life of older Turkish and Moroccan immigrants. The Journals of Gerontology, Series B: Psychological Sciences and Social Sciences, 75(5), 1113–1123. 10.1093/geronb/gbz024 30816945 PMC7161371

[CIT0022] Lager, D., Van Hoven, B., & Meijering, L. (2012). Places that matter: Place attachment and well-being of older Antillean migrants in the Netherlands. European Spatial Research and Policy, 19(1), 81–94. 10.2478/v10105-012-0007-6

[CIT0023] Lawton, M. P., & Nahemow, L. (1973). Ecology and the aging process. In C. Eisdorfer & M. P. Lawton (Eds.), The psychology of adult development and aging (pp. 619–674). American Psychological Association. 10.1037/10044-020

[CIT0024] Levasseur, M., Généreux, M., Bruneau, J.-F., Vanasse, A., Chabot, E., Beaulac, C., & Bédard, M.-M. (2015). Importance of proximity to resources, social support, transportation and neighborhood security for mobility and social participation in older adults: Results from a scoping study. BMC Public Health, 15(1), 503. 10.1186/s12889-015-1824-0 26002342 PMC4460861

[CIT0025] Meeks, S. (2020). Common themes for im/migration and aging: Social ties, cultural obligations, and intersectional challenges. Gerontologist, 60(2), 215–218. 10.1093/geront/gnz193 32092143

[CIT0026] Meeks, S. (2022). Age-friendly communities: Introduction to the special issue. Gerontologist, 62(1), 1–5. 10.1093/geront/gnab163 35029660

[CIT0027] Ministry of Health, Welfare and Sport. (2018). *Programma langer thuis [Longer at home program]*. https://open.overheid.nl/documenten/ronl-65f395b3-ac08-488d-8b3c-6eb8029ef450/pdf

[CIT0028] Mulliner, E., Riley, M., & Maliene, V. (2020). Older people’s preferences for housing and environment characteristics. Sustainability, 12(14), 5723. 10.3390/su12145723

[CIT0029] Nieboer, A. P., & Cramm, J. M. (2022). Age-friendly communities and well-being realization among older native and immigrant populations in the Netherlands: A theory-guided study protocol. BMC Geriatrics, 22(1), 273. 10.1186/s12877-022-02880-4 35366821 PMC8976267

[CIT0030] Omlo, J., Wolfers, M., & Stam, B. (2016). Betekenis van het ouder worden onder Marokkaanse en Turkse ouderen [Meaning of aging among Moroccan and Turkish older adults]. Municipality of Rotterdam. https://onderzoek010.nl/document/Betekenis-van-het-ouder-worden-onder-Marokkaanse-en-Turkse-ouderen/

[CIT0031] Pot, A., Keijzer, M., & De Bot, K. (2020). The language barrier in migrant aging. International Journal of Bilingual Education and Bilingualism, 23(9), 1139–1157. 10.1080/13670050.2018.1435627

[CIT0032] Schans, D. (2008). ‘They ought to do this for their parents’: Perceptions of filial obligations among immigrant and Dutch older people. Ageing & Society, 28(1), 49–66. 10.1017/S0144686X07006307

[CIT0033] Schellingerhout, R. (2004). Gezondheid en welzijn van allochtone ouderen [Health and well-being of older immigrants]. Sociaal en Cultureel Planbureau. https://repository.scp.nl/handle/publications/1041

[CIT0034] Schmolck, P., & Atkinson, J. (2002). PQ method software 2.11.

[CIT0035] Spring, A. (2018). Short-and long-term impacts of neighborhood built environment on self-rated health of older adults. Gerontologist, 58(1), 36–46. 10.1093/geront/gnx119 28958029 PMC5881656

[CIT0036] Statistics Netherlands. (2022). *Mensen met herkomst buiten Nederland wonen relatief vaak in grote steden* [*People from outside the Netherlands relatively often live in big cities*]. https://www.cbs.nl/nl-nl/nieuws/2022/48/mensen-met-herkomst-buiten-nederland-wonen-relatief-vaak-in-grote-steden

[CIT0037] Stock, F. (2014). Speaking of home: Home and identity in the multivoiced narratives of descendants of Moroccan and Turkish migrants in the Netherlands [Thesis fully internal (DIV)]. University of Groningen.

[CIT0038] Ten Kate, R. L., Bilecen, B., & Steverink, N. (2020). A closer look at loneliness: Why do first-generation migrants feel more lonely than their native Dutch counterparts? Gerontologist, 60(2), 291–301. 10.1093/geront/gnz192 31944240 PMC7039375

[CIT0039] United Nations Department of Economic and Social Affairs, Population Division. (2019). *International migration 2019: Report* (ST/ESA/SER.A/438). https://www.un.org/en/development/desa/population/migration/publications/migrationreport/docs/InternationalMigration2019_Report.pdf

[CIT0040] Van Cauwenberg, J., De Donder, L., Clarys, P., De Bourdeaudhuij, I., Buffel, T., De Witte, N., Dury, S., Verté, D., & Deforche, B. (2014). Relationships between the perceived neighborhood social environment and walking for transportation among older adults. Social Science & Medicine (1982), 104, 23–30. 10.1016/j.socscimed.2013.12.016 24581058

[CIT0041] van der Greft, S., & Fortuijn, J. D. (2017). Multiple disadvantage of older migrants and native Dutch older adults in deprived neighbourhoods in Amsterdam, the Netherlands: A life course perspective. GeoJournal, 82(3), 415–432. 10.1007/s10708-015-9691-x

[CIT0042] Van der Greft, S., Musterd, S., & Thissen, F. (2016). Residential dynamics and neighbourhood conditions of older migrants and native Dutch older adults in Amsterdam, The Netherlands. Ageing & Society, 36(1), 189–218. 10.1017/s0144686x14001159

[CIT0043] van der Star, M., Jong, A., & Manting, D. (2021) Vestigingspatronen van recente immigranten [Settlement patterns of recent immigrants]. Publication 4609. Planbureau voor de leefomgeving. https://www.pbl.nl/publicaties/vestigingspatronen-van-recente-immigranten

[CIT0044] Van Dijk, H. M., Cramm, J. M., Van Exel, J., & Nieboer, A. P. (2015). The ideal neighbourhood for ageing in place as perceived by frail and non-frail community-dwelling older people. Ageing & Society, 35(8), 1771–1795. 10.1017/s0144686x14000622

[CIT0045] van Hoof, J., van den Hoven, R. F., Hess, M., van Staalduinen, W. H., Hulsebosch-Janssen, L. M., & Dikken, J. (2022). How older people experience the age-friendliness of The Hague: A quantitative study. Cities, 124, 103568. 10.1016/j.cities.2022.103568

[CIT0046] Van Triest, N., & Van Vliet, J. (2017). De seniorvriendelijke stad [The age-friendly city]. Geron, 19, 44–47. 10.1007/s40718-017-0032-7

[CIT0047] Verkaik, R., Menting, J., & Mariën, V. (2019). Ervaren knelpunten van thuiswonende ouderen: Een onderzoek vanuit mensenrechtelijk perspectief [Perceived bottlenecks of community-dwelling older people: A study from a human rights perspective]. Nivel. https://www.nivel.nl/nl/publicatie/ervaren-knelpunten-van-thuiswonende-ouderen-een-onderzoek-vanuit-mensenrechtelijk

[CIT0048] Vos, E., Proper, K., Hilderink, H., Beek, A. v. d., & Bruin, S. d. (2021). Werkende mantelzorgers van ouderen. Verkenning van hun toekomst en ondersteuningsbehoeften [Working informal carers for older adults. Exploring their future and support needs]. Rijksinstituut voor Volksgezondheid en Milieu. https://www.rivm.nl/publicaties/werkende-mantelzorgers-van-ouderen

[CIT0049] Wahl, H.-W., & Gerstorf, D. (2020). Person–environment resources for aging well: Environmental docility and life space as conceptual pillars for future contextual gerontology. Gerontologist, 60(3), 368–375. 10.1093/geront/gnaa006 32240292

[CIT0050] Wahl, H. W., & Weisman, G. D. (2003). Environmental gerontology at the beginning of the new millennium: Reflections on its historical, empirical, and theoretical development. Gerontologist, 43(5), 616–627. 10.1093/geront/43.5.616 14570958

[CIT0051] Watts, S., & Stenner, P. (2012). Doing Q methodological research. Theory, method and interpretation. Sage Publications Ltd. 10.4135/9781446251911

[CIT0052] Witter, Y., & Fokkema, T. (2018). Huisvesting en zorg voor oudere migranten in Nederland [Housing and care for older migrants in the Netherlands]. Demos: Bulletin over Bevolking en Samenleving, 34(6), 1–4. https://nidi.nl/wp-content/uploads/2019/11/demos-34-06.pdf

[CIT0053] World Health Organization. (2007). *Global age-friendly cities: A guide*. https://iris.who.int/bitstream/handle/10665/43755/9789241547307_eng.pdf?sequence=1

