# Supplementary material for: Needs for Aging in Place: Views of Older Moroccan Adults in the Netherlands
Source: Gerontologist. 2023 Nov 6;64(7):gnad154. doi: 10.1093/geront/gnad154 (PMC11190963; doi:10.1093/geront/gnad154)
Supplement: gnad154_suppl_Supplementary_Material [file gnad154_suppl_supplementary_material.docx]

**Online Supplementary Material**

**Section 1. Distribution of Study Participants in the Four Largest Cities in the Netherlands** (*n* = 30)

**
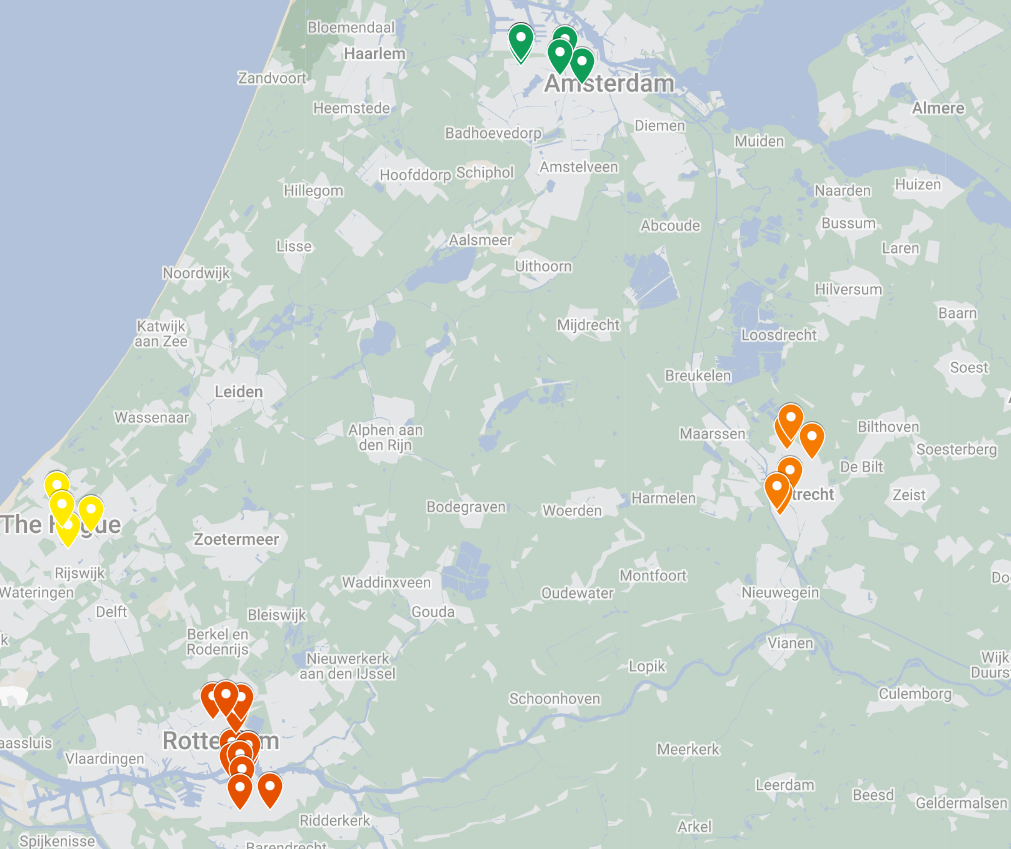
**

**Section 2. Details of Q-Set Development**

In Q methodological research, the Q set must include statements that cover all relevant aspects of the topic of interest (Watts & Stenner, 2012). First, a review of the most recent literature on community age-friendliness was performed. Torku et al.’s (2021) systematic review of age-friendly cities and communities was used to identify studies of age-friendliness (e.g., Liddle et al., 2014; Orpana et al., 2016; Wong et al., 2017). Additional articles (e.g., Atkins, 2019; Dikken et al., 2020; Van Dijk et al., 2015) were also studied. The checklists of the Modified Community Assessment Survey for Older Adults (Dellamora, 2013) and the World Health Organization’s (WHO’s; 2007) global age-friendly cities guide were consulted. This process led to the collection of 537 (overlapping) statements, which were allocated to the WHO’s eight domains encompassing the characteristics of the physical and social environments and services needed for active aging (WHO, 2007) and older adults’ health and well-being (WHO, 2015). In the second phase of the process, the researchers jointly discussed the statements list and individually created concise and comprehensive lists with manageable numbers of statements. The researchers then merged these lists with the removal of overlap to yield the Q set. Special consideration was given to ensuring that a sufficient number of statements fell under each domain and that relevant resources were covered.

The Q statements, developed in Dutch, were translated into Arabic and Turkish by a professional translation agency. To ensure the clarity, comprehensiveness, and representativeness of the Q set for older adults residing in the Netherlands, 19 pilot interviews were conducted with older Dutch natives and older migrants with Moroccan, Turkish, and Surinamese backgrounds. Six of these interviews were conducted with older Moroccans in Dutch, Arabic, or Berber, according to the interviewees’ preference and/or language proficiency. The focus in these interviews was to identify any missing statements about relevant neighborhood resources that are important for older (migrant) adults’ aging in place. We asked the interviewees to perform the sorting procedure, then asked whether any statements were missing and/or whether they would like to add any relevant neighborhood aspects. During the pilot period, the statements underwent four rounds of changes. Statement 38 (“Opportunities for sports in the neighborhood”) was added, and “cultural” was added to statement 16 (“A neighborhood where social/cultural activities are organized”). Fifteen other statements were reformulated to increase clarity, e.g., statement 25 (“Interactions between young and older adults”) was reformulated to (“Contact between young and old in the neighborhood”). We continued the pilot interviews until several consecutive participants had no further points to add. The pilot interviews ensured that the Q set was representative, the statement wording and translations were clear for older (migrant) adults, and the sorting procedure was feasible, especially for older migrant adults. We also asked participants interviewed for the main study whether any neighborhood aspects were missing; no participant responded in the affirmative. We are thus confident that the Q set is representative and can be used to obtain interviewees’ views on the relative importance of neighborhood resources for aging in place.

**Section 3. English Translation of the Interview Guide and Q-Sort Instructions**

**General Introduction:**

Thank you for participating in this research. My name is _______ and I am conducting this study on behalf of Erasmus University Rotterdam. I am interested in understanding how you can age comfortably in your own neighborhood. For this research, we will be interviewing a number of people with different experiences and perspectives on their neighborhood. We will go through a set of statements, which will take approximately half an hour. Afterward, I will ask you some questions regarding your perspectives. If you have any questions now or during the study, please let me know. All information will be anonymized and solely used for research purposes. Are you in agreement with this?

Identifier participant

**Background Information**

**Step 1 (this session has to be recorded, ask for consent/permission to record)**

This set of cards consists of statements about what you find important in your neighborhood. I will ask you later how you perceive these statements: Some of these statements may be very important to you in order to feel good in your neighborhood, while others may be less important. It is about what you personally find important, not whether it is already the case in your neighborhood. There are no right or wrong answers, and we will go through it step by step.

To get a sense of the statements, I would like you to read each card while thinking about which aspects are important to you in the neighborhood. Then, place the cards into three piles: important, not (or least) important, or neutral/don't know. Don't worry about the placement for now. Later, I will ask you to assess them more precisely, and you will have the opportunity to change their placement if you wish to do so.

*Give the respondent as much time as needed.*

*Please note the number of cards in each pile on the response sheet.*

**Step 2**

**IMPORTANT Statements**

Take the pile of statements that you consider most important and select the two statements that you believe are the most important. Place these statements in the rightmost column (indicate).

Go back to the remaining pile and select three statements that closely align with your perspective. Place these three statements in the second rightmost column (indicate).

Continue placing the remaining statements toward the center until this pile of cards is exhausted.

**NOT/LEAST IMPORTANT Statements**

Now, we will do the same with the statements that you consider not important or least important. So, take the pile of statements that you do not find important or find least important, and select the two statements that you believe are the least important. Place these statements in the leftmost column (indicate).

Go back to the remaining pile and select the three statements that least align with your perspective. Place these three statements in the second leftmost column (indicate).

Continue placing the remaining statements toward the center until this pile of cards is exhausted.

**NEUTRAL/DON’T KNOW Statements**

Now, take the remaining pile of statements. Take some time to review them once again, and place the statements in the remaining open spaces of the response sheet, wherever you think they should be placed. Trust your judgment in determining their appropriate placement.

*Once all the cards have been placed, ask: Are you satisfied with how you have placed the cards? Or are there a few that you would like to change?*

*If the respondent is content, record the order of the cards on the response sheet.*

**Questions regarding the second step**

1. If you were to summarize what is important for you to feel good in your neighborhood, what would you say?

Follow-up questions:

- What were you thinking when you placed the cards?
- What influenced your answers? Is there anything else you would like to mention?

1. If you were to summarize what is not important or least important for you to feel good in your neighborhood, what would you say?

Follow-up questions:

- What were you thinking when you placed the cards?
- What influenced your answers? Is there anything else you would like to mention?

1. Which cards did you place all the way to the right?
2. You indicated that it is important for you to feel good in the neighborhood that... (read the first card) ... Why did you place this card in this position?

Follow-up questions:

- Can you elaborate more on this statement? Why does it contribute to your sense of well-being in the neighborhood?
- What factors contribute to your strong feelings about this statement?
- Also, inquire about specific terms on the card: What does safety mean to you? What does respect mean? What specific amenities or facilities are important to you?

1. You indicated that it is important for you to feel good in the neighborhood that... (read the second card) ... Why did you place this card in this position?

Follow-up questions:

- Can you elaborate more on this statement?
- Why does this contribute to your sense of well-being in the neighborhood?
- What factors contribute to your strong feelings about this statement?
- Also, inquire about specific terms on the card: What does safety mean to you? What does respect mean? What specific amenities or facilities are important to you?

1. Which cards did you place in the second rightmost column?
2. You indicated that it is important for you to feel good in the neighborhood that... (read the first card) ... Why did you place this card in this position?

Follow-up questions:

- Can you elaborate more on this statement?
- Why does this contribute to your sense of well-being in the neighborhood?
- What factors contribute to your strong feelings about this statement?
- Also, inquire about specific terms on the card: What does safety mean to you? What does respect mean? What specific amenities or facilities are important to you?
- **Ask these questions for the entire column!**

1. Which cards did you place all the way to the left?
2. You indicated that it is not important or least important for you to feel good in the neighborhood that... (read the first card) ... Why did you place this card in this position?

- Follow-up questions:

- Can you elaborate more on this statement?
- Why does this contribute to your sense of well-being in the neighborhood?
- What factors contribute to your strong feelings about this statement?
- Also, inquire about specific terms on the card: What does safety mean to you? What does respect mean? What specific amenities or facilities are important to you?

1. You indicated that it is not important or least important for you to feel good in the neighborhood that... (read the second card) ... Why did you place this card in this position?

Follow-up questions:

- Can you elaborate more on this statement?
- Why does this contribute to your sense of well-being in the neighborhood?
- What factors contribute to your strong feelings about this statement?
- Also, inquire about specific terms on the card: What does safety mean to you? What does respect mean? What specific amenities or facilities are important to you?

1. Which cards did you place in the second leftmost column?
2. You indicated that it is not important or least important for you to feel good in the neighborhood that... (read the first card) ... Why did you place this card in this position?

Follow-up questions:

- Can you elaborate more on this statement?
- Why does this contribute to your sense of well-being in the neighborhood?
- What factors contribute to your strong feelings about this statement?
- Also, inquire about specific terms on the card: What does safety mean to you? What does respect mean? What specific amenities or facilities are important to you?
- **Ask these questions for the entire column!**

1. Is there a statement that you feel is missing and would like to add? If so, why would you like to include it?
2. In conclusion, could you summarize in two or three sentences what is important for you to feel good in the neighborhood?
3. Do you know someone (with a different perspective) who would be willing to participate in the interview?

**Section 4. Details of the Ranking Procedure**

Participants’ initial sorting of the Q statements into three piles allowed them to formulate initial impressions and become familiar with the statements, facilitating decision making in the second step; it was provisional and aided the construction of the final Q sort (Watts & Stenner, 2012). We also believe that this step was especially important for older (Moroccan) adults, who may need more time to read (or hear) all of the statements and formulate judgements of their relative importance. Participants’ ability to see (hear) all statements in each of the three piles at once was especially important, as the statements must be rank-ordered relative to other statements (Watts & Stenner, 2012). In the second step, participants were asked to perform rank-ordering on the sorting grid (Figure 1), beginning with the statements they found to be most important (highest ranking, right end of the grid) and proceeding through the continuum to the statements that they found to be least important (lowest ranking, left end of the grid).

**Section 5. Details of Viewpoint Interpretation**

To interpret each factor, the authors conducted a holistic exploration of statement patterning within and between the factors/viewpoints. Characterizing, distinguishing, and consensus statements (Van Exel & De Graaf, 2005) were given special attention. Characterizing statements are those ranked at both extremes of the sorting grid, and helped to produce initial viewpoint descriptions. Distinguishing (with rankings differing significantly from those in other factors) and consensus (with similar rankings in all factors) statements were further consulted to highlight differences and similarities among viewpoints (Berghout et al., 2015; Van Exel & De Graaf, 2005).

The authors used the “crib sheet” method introduced by Watts and Stenner (2012), starting with the characterizing statements. Cross-factor item comparisons were then performed to explore statements ranking higher and lower than in other factors, thereby identifying potentially important statements ranked toward the middle (zero point) of the distribution. Watts and Stenner (2012) emphasized that such statement rankings are of potential importance when examined in relation to rankings in other factors and warned against the tendency to directly assume their neutrality. Qualitative data on all participants significantly loading on each factor (factor exemplars) were studied extensively to improve the understanding and description of the viewpoint. The participants’ own words were used to further clarify their preferences and the reasoning underlying their choices (Hackert et al., 2019; Van Exel & De Graaf, 2005).

To identify the Q sorts of participants loading significantly (*p* < 0.05) on a specific factor, the following formula was used: 1.96 × (1 / √n), where *n* is the number of statements in the Q set (Watts & Stenner, 2005, 2012). Participants whose Q sorts had factor loadings of a minimum of ±0.32 with no confounding (i.e., the square of loading on that factor exceeded the sum of squares of the Q-sort loadings on the other three factors) were considered to be factor exemplars. Q sorts that were confounded (i.e., loading significantly on two factors) or did not load significantly on any of the four factors were not included in factor interpretation (Hackert et al., 2019; Watts & Stenner, 2005, 2012).

**References**

Atkins, M. T. (2019). Creating age-friendly cities: Prioritizing interventions with Q-methodology. *International Planning Studies*, 25(4), 303–319. <https://doi.org/10.1080/13563475.2019.1608164>

Berghout, M., Van Exel, J., Leensvaart, L., & Cramm, J. M. (2015). Healthcare professionals' views on patient-centered care in hospitals. *BMC Health Services Research*, 15, 1–13.

<https://doi.org/10.1186/s12913-015-1049-z>

Dellamora, M. (2013). How age friendly is this city? Strategies for assessing age-friendliness. MS thesis, Health and Rehabilitation Sciences, Western University.

Dikken, J., van den Hoven, R. F., van Staalduinen, W. H., Hulsebosch-Janssen, L. M., & van Hoof, J. (2020). How older people experience the age-friendliness of their city: Development of the age-friendly cities and communities questionnaire. *International Journal of Environmental Research and Public Health*, 17(18), 6867. <https://doi.org/10.3390/ijerph17186867>

Hackert, M. Q., Brouwer, W. B., Hoefman, R. J., & van Exel, J. (2019). Views of older people in the Netherlands on wellbeing: A Q-methodology study. *Social Science & Medicine*, 240, 112535. <https://doi.org/10.1016/j.socscimed.2019.112535>

Liddle, J., Scharf, T., Bartlam, B., Bernard, M., & Sim, J. (2014). Exploring the age-friendliness of purpose-built retirement communities: Evidence from England. *Ageing & Society*, 34(9), 1601–1629. <https://doi.org/10.1017/S0144686X13000366>

Orpana, H., Chawla, M., Gallagher, E., & Escaravage, E. (2016). Developing indicators for evaluation of age-friendly communities in Canada: Process and results. *Health Promotion and Chronic Disease Prevention in Canada: Research, Policy and Practice*, 36(10), 214. <https://doi.org/10.24095/hpcdp.36.10.02>

Torku, A., Chan, A. P. C., & Yung, E. H. K. (2021). Age-friendly cities and communities: A review and future directions. *Ageing & Society*, 41(10), 2242–2279. <https://doi.org/10.1017/S0144686X20000239>

Van Dijk, H. M., Cramm, J. M., Van Exel, J., & Nieboer, A. P. (2015). The ideal neighbourhood for ageing in place as perceived by frail and non-frail community-dwelling older people. *Ageing & Society*, 35(8), 1771–1795. <https://doi.org/10.1017/S0144686X14000622>

Van Exel, J., & De Graaf, G. (2005). *Q methodology: A sneak preview*. [available from [www.jobvanexel.nl](http://www.jobvanexel.nl)].

Watts, S., & Stenner, P. (2005). Doing Q methodology: Theory, method and interpretation. *Qualitative Research in Psychology*, 2(1), 67–91. <https://doi.org/10.1191/1478088705qp022oa>

Watts, S., & Stenner, P. (2012). Doing Q methodological research. Theory, method and interpretation. SAGE Publications Ltd. <https://doi.org/10.4135/9781446251911>

Wong, M., Yu, R., & Woo, J. (2017). Effects of perceived neighbourhood environments on self-rated health among community-dwelling older Chinese. *International Journal of Environmental Research and Public Health*, 14(6), 614. <https://doi.org/10.3390/ijerph14060614>

World Health Organization. (2007). *Global age-friendly cities: A guide*. https://iris.who.int/bitstream/handle/10665/43755/9789241547307_eng.pdf?sequence=1

World Health Organization. (2015). *Measuring the age-friendliness of cities: A guide to using core indicators*. <https://apps.who.int/iris/handle/10665/203830>.
